# European Correspondence

**Published:** 1878-12

**Authors:** R. J. Nunn

**Affiliations:** Paris


### EUROPEAN CORRESPONDENCE.

PARIS, September 30, 1878.

Of all the curious and interesting discoveries, or, perhaps, more correctly speaking, rediscoveries of modern times, probably the most wonderful is the metalloscopy of Dr. Burg, which has been undergoing investigation for some time by Prof. Charcot and others at the Salpetriere.

The facts demonstrated evidence once more the truth of phenomena which have been, I may almost say are, regarded as spurious, such as mesmerism, biology, etc., whose discoveries were scouted and reviled during their lives, and even now their followers are by the public at least looked upon almost as cheats, and swindlers, or else as fools, enthusiasts, or monomaniacs; as persons to be investigated, not believed. All this, and much more even, not to make mention of the accusations against them, originating in the pulpit or being in close connection with his Satanic Majesty. But Mesmor is no longer a fool or a humbug, nor are Perkins' tractors a fraud. Mesmor is amply revenged, and it is not a little curious that his justification should come from the very city from which came the most scathing denunciation of his discovery. Nor is this the first time that the truth demonstrated by Mesmor have been again brought to light, and in Paris too, without the least acknowledgement to him. It is not many years since M. Velpean brought forward the same thing, and ungenerously called it forsooth not mesmorism, but hypurtism.

Metalloscopy is the investigation of the same phenomena extended over a far wider range as regards effects, but confined to one class of individuals, namely: Hystoro-epileptics; in other words, it is the over-sensitiveness which may exist in a peculiar class of nervous patients to certain electrical conditions or to the sensations of certain substaces somewhat like the exaggerated sensitiveness to light—photographic—which exists in certain ophthalmic affections. But if this sensitiveness becomes so remarkable in diseased conditions, certainly it is but fair to conclude that it exists in a dormant, or more probably in an inappreciable condition in each individual, becoming exagerated or tangible, so to speak, only in peculiar organizations, or under the influence of certain morbid conditions.

But let the causes and the explanation be what they may, the facts, the phenomena, remain; and the time has come when the popular and scientific judgment—passed as has always been the case on such subjects hastily and without just and fair investigation—upon all this class of nervous phenomena from witchcraft to spiritualism, from ecstasy to metalloscopy, should be revived. That the mysterious influence which gives to our living organism a certain control more or less powerful over another creature, which appears even to extend to the influence of organized beings over inorganic matter, and by which, as now demonstrated, inorganic matter reacts upon the living tissues, and which appears to be another form or property of that ether which unites the universe into one whole, should be reinvestigated patiently and truthfully, and not with bias or partisan spirit. It is high time that philosophers should be worthy of the name; that they should not be ashamed to acknowledge their ignorance, or their inability to fathom all the secrets of nature. Whenever upon new lines of investigation presented to them they have ventured to pass judgment, giving an opinion based upon their past experience only, almost invariably have they been mistaken, and so they ever will be until they have learned to say and to apply the words, "I do not know."

This is why, in this "old world," new truths take root but slowly. Ignorance and philosophy both join in opposing them, and both, strange to say, for the same reason—vanity; for the most ignorant individual always thinks himself right, and adheres to his opinion with the greatest obstinacy, while the philosopher is never prepared to abandon a pet theory, even after it has been proven erroneous. If the teaching of humility could be made a portion of the wave of modern education—so called—now burdening the world, it would be of much more benefit to posterity than much of the airs and misnamed accomplishments at present taught.

As metalloscopy has only been investigated so far in cases of hystero-epilepsy, a short description of this disease may not be out of place for the benefit of those who desire to repeat the experiments when opportunities offer. It may enable them to distinguish between this disease and true epilepsy.

Hystero-epilepsy is accompanied by tenderness of one or both ovaries. There is loss of sensitiveness of one side of the body, rarely of both, and there is unilateral diminution of muscular power. Color blindness may or may not be present. The perception of violet is generally first lost, while red is the most persistent. There are attacks of epileptiform convulsions, which have in each patient a definite form, but in the main it has been said to consist of three stages: 1st. An epileptiform stage; 2d. Stage of violent movements ("*phase des grande mouvements*"); 3d. Emotional stage ("*phase des attitudes passionelles*"). A point of differential diagnosis between this disease and true epilepsy insisted upon by Prof. Charcot is, that while in hystero-epilepsy the temperature during the paroxysm is rarely as high as 100°, during that of true epilepsy it may rise as high as 107°. Through the action of magnetic current, electrical currents, electrolized plate, strictly unipolar galvanic applications, simple metallic plates of various kinds, according to the metallic sensitiveness of the patient, solenoids, etc., there is a transference of the unilateral symptoms from one side of the body to the other. The disease occurs generally during the menstruating period of life—rarely before it or after it. Recoveries are not common. The epileptiform convulsions can be shortened by pressure on the diseased ovary. The mesmeric and also the cataleptic conditions are often readily induced in these patients. There is generally a zone of peripheral tenderness, by irritating which the epileptiform convulsions may be brought on. When the metallic sensitiveness has been once ascertained by external applications, the internal administration of the same metal will certainly benefit if it does not cure the patient. After the amelioration of the symptoms by the internal administration of the appropriate metal, the external application may still produce the same phenomena as at first, but more slowly, and this will be the case as long as the cure is incomplete.

It must be remarked that physicians can be easily deceived in the course of their examinations, for hysteria is almost a chamelion disease, modifying its appearances according to the changes of surrounding circumstances, and the therapeutic means employed to remedy it; nor is it at all probable that the analysis of hysterical affection by means of metalloscopic investigations will soon be popularized, because the amount of time, patience and careful observation required for such examinations would be far too great a tax upon the time of the ordinary, hurried "general practitioner," and as there does not appear to be much immediate prospect of remedying this evil, this mode of investigation is likely for the present, at least, to be little more than a scientific curiosity, or to remain for some time in the hand of specialists.

Of course a detailed account of these experiments would be quite impossible in a rambling letter like the present, and, therefore, a description of the leading feature is all that has been attempted, at the same time a hint as to one or two of the difficulties which may be encountered or the illusions which may mislead the inexperienced investigator may not be misplaced. "Expectant attention" holds the first place; the word is already too well known to need a lengthy definition. In cases otherwise quite reliable, the physician must avoid giving the least hint, either to the patient or her friends, of the character of the phenomena or symptoms to be looked for, and the exercise of a like prudent reticence may enable the investigator to avoid another source of error, viz: the counterfeiting of the phenomena by interested persons.

Upon closely examining these curious phenomena, it will be observed that—

1. In certain cases of hystero epilepsy one of the causes of the disease appears to be the absence of one or more chemical principles from the system.

2. The external application of such substance produces seemingly certain phenomena, through which the substance required may be recognized.

3. The internal exhibition of this substance, while curing the disease, diminishes "pari passu" the external sensitiveness to the substance.

4. In certain cases the symptoms appear to be intimately associated with a more or less inflamed ovary.

In this class of cases, failing other remedies, would not the extirpation of the diseased ovary be indicated? Would it not be justifiable? R. J. NUNN.

